# Towards Circular Water Treatment: Adsorption Mechanism and Analytical Characterization of Metformin Retention on Amberlite XAD7HP Resin

**DOI:** 10.3390/polym18141751

**Published:** 2026-07-17

**Authors:** Valentin Romeo Marin, Nicoleta Mirela Marin, Toma Galaon, Adriana Mariana Borș, Ludmila Motelica, Otilia Ruxandra Radacina, Marian Rascov, Ovidiu Oprea

**Affiliations:** 1 C.M.V. DR. MARIN ROMEO SRL, 235601 Oraş Scorniceşti, Romania; romeomarinvet@yahoo.com; 2 National Research and Development Institute for Industrial Ecology ECOIND, 060652 Bucharest, Romania; tomagalaon@yahoo.com; 3 Department of Analytical and Physical Chemistry, University of Bucharest, 030018 Bucharest, Romania; 4 Department of Oxide Materials Science and Engineering, National University of Science and Technology POLITEHNICA Bucharest, 060042 Bucharest, Romania; otiliaradacina@yahoo.com (O.R.R.); marian.rascov@yahoo.com (M.R.); 5 National Institute for R&D for Optoelectronics—Subsidiary, Research Institute for Hydraulics and Pneumatics—INOE 2000-IHP, 040558 Bucharest, Romania; bors.ihp@fluidas.ro; 6 Research Center for Advanced Materials, Products and Processes, National University of Science and Technology POLITEHNICA Bucharest, 060042 Bucharest, Romania; ludmila.motelica@upb.ro; 7 National Centre for Micro- and Nanomaterials, National University of Science and Technology POLITEHNICA Bucharest, 060042 Bucharest, Romania; ovidiu.oprea@upb.ro; 8 Academy of Romanian Scientists, 050045 Bucharest, Romania; 9 Faculty of Chemical Engineering and Biotechnologies, National University of Science and Technology POLITEHNICA Bucharest, 011061 Bucharest, Romania

**Keywords:** metformin, amberlite XAD7HP, adsorption, kinetics, desorption experiments

## Abstract

This study evaluates the adsorption, structural characterization, and regeneration performance of acrylic resin Amberlite XAD7HP (X7) for the removal of metformin (MET), an emerging pharmaceutical contaminant. MET concentrations after adsorption were quantified using the linear UV–Vis method at 232 nm (R^2^ = 0.9998). Adsorption kinetics followed the pseudo-second-order model (R^2^ =0.9881), and equilibrium data fitted the Langmuir isotherm, confirming monolayer adsorption. Desorption experiments showed that acidic media were ineffective (<10%), whereas the mixed (1:1) MeOH–1M HCl system achieved 89.3% MET recovery, enabling efficient resin desorption. FTIR confirmed MET retention through attenuation of N–H stretching bands at 3360–3290 cm^−1^, the shift of the C=N vibration from 1628 cm^−1^ to 1605 cm^−1^, and the appearance of a new band at 1542 cm^−1^, indicating hydrogen bonding and dipole–dipole interactions with the resin. SEM micrographs revealed a clear transition from the X7, rough morphology to a smoother, partially occluded surface after adsorption, consistent with pore filling by MET. EDX analysis further confirmed MET uptake through the appearance of a distinct N signal and increased O content, serving as elemental markers of drug adsorption. TG/DSC demonstrated enhanced thermal stability and modified decomposition profiles for the resin loaded with MET, while XRD patterns confirmed the amorphous nature of X7 and the absence of crystalline MET deposits, indicating molecular-level dispersion. The integrated analytical, structural, kinetic, and desorption results highlight the potential of desorbed acrylic resin as a sustainable material for mitigating pharmaceutical pollution in aquatic environments.

## 1. Introduction

The continuous detection of pharmaceutical residues in aquatic systems has intensified global concern regarding their persistence, transformation pathways, and potential ecotoxicological effects [1,2,3,4,5,6,7,8,9]. Within this class of contaminants [10,11,12,13,14,15], metformin—a highly polar and hydrophilic biguanide used at multi-tone annual scales—stands out as one of the most frequently identified compounds in influent, effluent, and surface waters [16,17,18,19,20,21,22,23,24,25]. MET (1,1-dimethylbiguanide hydrochloride), the leading oral antidiabetic medication worldwide, acts primarily through activation of AMP-activated protein kinase (AMPK), thereby reducing hepatic gluconeogenesis and improving peripheral insulin responsiveness [26,27]. Its extensive therapeutic use, combined with limited removal during conventional wastewater treatment, has resulted in its recurrent detection in natural waters, raising concerns about environmental persistence and potential ecological risks [28,29,30,31,32].

Its limited biodegradability, near-complete excretion in its unmetabolized form, and resistance to conventional wastewater treatment processes have positioned MET as a priority emerging pollutant in environmental monitoring programs [33,34,35,36]. Furthermore, the formation of transformation products such as guanylurea, whose toxicity remains insufficiently characterized, underscores the need for robust removal strategies [16,18,29,37,38,39,40,41,42].

A wide range of technologies has been explored for drug mitigation [43,44], including advanced oxidation processes (AOPs) [45,46,47,48,49], electrochemical degradation [50], membrane filtration [51], and adsorption using carbonaceous [23] or hybrid materials [52]. While AOPs can achieve high degradation efficiencies, they often require elevated energy input and may generate secondary by-products. Membrane-based processes provide excellent retention but suffer from fouling and high operational costs. Adsorption has therefore emerged as a cost-effective and scalable alternative, yet the literature reveals substantial divergence regarding the dominant mechanisms governing MET uptake. Some studies attribute retention to electrostatic attraction between protonated MET and negatively charged surfaces, whereas others report significant contributions from hydrogen bonding, dipole–dipole interactions, or donor–acceptor orbital coupling, reflecting the structural complexity of the biguanide moiety and the variability of adsorbent surface chemistries [53,54,55,56,57,58,59,60].

Despite extensive research on activated carbons, biochars, and functionalized inorganic–organic hybrids, nonionic acrylic resins remain largely underexplored for pharmaceutical removal [61,62,63,64]. Among them, X7 is a macroporous acrylic ester material with high surface area and a polar ester-based matrix that provides selectivity toward hydrophilic organic molecules [65,66,67]. These structural characteristics make it a promising adsorbent for the retention and controlled release of pharmaceuticals such as MET.

This gap is notable given the resin’s macroporous architecture, moderate polarity, and abundance of ester carbonyl groups, which theoretically enable strong interactions with protonated MET species across environmentally relevant pH ranges. Moreover, the potential for efficient regeneration and solvent-assisted desorption positions acrylic resins as promising candidates for circular water treatment frameworks—an aspect insufficiently addressed in current research.

To date, no comprehensive study has evaluated MET adsorption onto X7, nor correlated adsorption performance with advanced solid-state characterization techniques capable of elucidating molecular-level interaction mechanisms. This lack of mechanistic insight limits the rational design of polymeric adsorbents for highly polar pharmaceuticals. Given the growing concern over the environmental persistence of MET as a high-consumption pharmaceutical and representative emerging contaminant, the present study aims to investigate—for the first time in the scientific literature—the adsorption of MET onto the nonionic acrylic resin X7 by systematically evaluating the key operational parameters (pH, adsorbent dosage, contact time, and initial concentration), the kinetics and equilibrium isotherms, and the regeneration efficiency, while correlating these findings with advanced solid-state characterization techniques (FTIR, SEM/EDX, TG/DSC, and XRD) to elucidate the molecular-level interaction mechanisms governing the process.

## 2. Materials and Methods

### 2.1. Reagents

All chemicals used in this study were of analytical grade and purchased from Sigma-Aldrich (St. Louis, MO, USA), unless otherwise specified. MET hydrochloride (MET) was used as the target pharmaceutical compound, while Amberlite XAD7HP acrylic ester resin (DuPont/Dow Chemical) served as the adsorbent material. The following reagents were employed for pH adjustment and buffer preparation: hydrochloric acid (1 M HCl), sodium hydroxide (1 M NaOH), phosphate buffer solutions (pH 2.0 and 8.0), acetate buffer solutions (pH 4.2 and 6.0), and carbonate buffer (pH 10.2). Desorption studies were carried out using methanol (MeOH), ethanol (EtOH), acetone, and acetonitrile (AcCN), as well as 1:1 (*v*/*v*) mixtures of 1 M HCl with MeOH, EtOH, acetone, or AcCN. All aqueous solutions were prepared using ultrapure water.

### 2.2. UV–Vis Method

**Figure 1:**
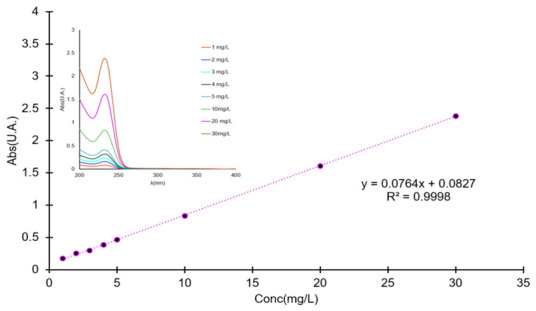
UV–Vis spectra of MET standard solutions (1–30 mg/L) and the corresponding calibration graph. Data represent mean values of duplicate experiments with a standard deviation below 3%.

The UV–Vis method used to quantify MET in the filtrates after adsorption on X7 showed excellent linearity and analytical reliability. MET exhibited a distinct absorption maximum at 232 nm in the 200–400 nm range, corresponding to the π→π* transition of the biguanide group; this wavelength was selected for quantitative analysis. The calibration curve constructed for 1–30 mg/L MET solutions displayed a strictly linear Beer–Lambert relationship, with the regression equation A_(232nm)_ = 0.0789C + 0.0512 and R^2^ = 0.9998 (Figure 1). These results confirm the method’s high sensitivity and suitability for monitoring residual MET concentrations during adsorption experiments.

### 2.3. Fourier-Transform Infrared Spectroscopy (FTIR)

FTIR spectra for X7, MET, and the MET-loaded X7 resin were recorded at room temperature using a Nicolet iS50R spectrometer (Thermo Fisher Scientific, Madison, WI, USA) equipped with an ATR module. The samples were placed directly onto the ATR crystal, and each spectrum was obtained by averaging 32 scans over the 4000–400 cm^−1^ range, with a spectral resolution of 4 cm^−1^.

### 2.4. TG–DSC

The thermal behavior of the X7, MET and X7-MET was examined using a NETZSCH STA 449C Jupiter simultaneous TG–DSC analyzer (NETZSCH-Gerätebau GmbH, Selb, Germany). Each sample was placed in an open alumina crucible and heated from room temperature up to 900 °C at a constant rate of 10 K/min, under a continuous flow of dried air (50 mL/min). An empty alumina crucible served as the reference during all measurements.

### 2.5. Scanning Electron Microscopy (SEM) and Energy-Dispersive X-Ray Spectroscopy (EDS)

The morphology and particle size of the MET, X7 and X7-MET samples were investigated by scanning electron microscopy (SEM). The analyses were performed using a Quanta Inspect F50 microscope (Thermo Fisher/FEI, Eindhoven, The Netherlands), equipped with a field emission electron source capable of reaching a resolution of approximately 1.2 nm. The system also includes an EDS detector with a resolution of 133 eV for the MnK line, allowing the identification of elements present on the surface. The powders were fixed on conductive carbon supports and introduced into the microscope chamber for examination. SEM micrographs were collected to highlight the specific morphology of each sample, the particle distribution and any microstructural changes induced by functionalization or MET loading. EDS analysis was used complementarily to confirm the elemental distribution on the surface and to compositionally differentiate the MET, X7 and X7-MET samples.

### 2.6. X-Ray Diffraction (XRD)

The structural characterization of the MET, X7, and X7-MET samples was carried out by X-ray diffraction using a MiniFlex 600C diffractometer (Rigaku, Tokyo, Japan) equipped with a Cu Kα radiation source (λ = 1.5406 Å). Diffraction patterns were collected in Bragg–Brentano geometry over the 2θ range of 10–80°, with a step size of 0.01° and a scanning rate of 1°·min^−1^. Phase identification was performed by comparing the experimental reflections with standard reference databases. The average crystallite size was estimated using the Scherrer equation applied to the main diffraction peaks. Unit cell parameters were determined by refining the positions of the dominant reflections using SmartLab Studio II software (version 4.4). This analysis enabled the assessment of structural modifications associated with the functionalization of X7 and the subsequent loading with MET.

### 2.7. Procedure for Studying the Kinetics of MET Adsorption onto X7

The adsorption kinetics of MET onto the X7 resin were investigated by monitoring the variation of MET concentration in solution as a function of contact time. Fixed amounts of X7 (0.05 g) were mixed with 0.01 L of MET solution with an initial concentration of 300 mg/L. The mixtures were maintained at 175 rpm and T = 25 ± 2 °C, at selected time intervals ranging from 10 to 90 min. At each contact time, the concentration of MET in the solution (C_t_) was determined by the UV–Vis method. The time required to reach adsorption equilibrium was defined as the minimum contact time at which no further change in MET concentration was observed. The quantity of MET adsorbed at time t, expressed per gram of X7 resin, was calculated using Equation (1) [68]:

(1) $$Q_{t}=\frac{(C_{i}-C_{t})V}{m}$$

where Q_t_ (mg/g) is the adsorption capacity at time t; C_i_ (mg/L) is the initial MET concentration; C_t_ (mg/L) is the concentration at time t; V (L) is the solution volume; and m (g) is the mass of resin.

### 2.8. Procedure for Studying the Influence of Adsorbent Dosage

A batch adsorption experiment was carried out to evaluate the effect of X7 dosage on MET removal from aqueous solutions. For each test, a fixed volume of MET solution with a known C_i_ was brought into contact with different masses of X7 resin in 100 mL Erlenmeyer flasks. The mixtures were agitated for 80 min at T = 25 ± 2 °C; then, the solid phase was separated by filtration and the supernatant was collected for analysis.

The C_e_ of MET in the liquid phase was determined by the UV–Vis method. The adsorption capacity Q_e_ (mg/g) and removal efficiency R (%) were calculated using Equations (2) and (3) [68,69].

(2) $$Q_{e}=\frac{(C_{i}-C_{e})V}{m}$$

(3) $$R(\%)=\frac{C_{i}-C_{e}}{C_{i}}\times 100$$

where C_e_ (mg/L) represents the MET concentration in solution at equilibrium after adsorption on X7.

### 2.9. Procedure for Studying the Influence of pH

The effect of pH on the adsorption of MET onto X7 was evaluated through batch experiments. MET solutions with fixed C_i_ = 200 mg/L were prepared in buffer solutions. The buffer solutions employed were: phosphate buffer for pH 2.0, acetate buffer for pH 4.2, acetate buffer for pH 6.0, phosphate buffer for pH 8.0, and carbonate buffer for pH 10.2. For each pH condition, 0.05 g of X7 resin was added to 10 mL of MET solution in 100 mL Erlenmeyer flasks. The samples were agitated at 175 rpm for 80 min at T = 25 ± 2 °C to ensure sufficient contact between the adsorbent and the solute. After equilibration, the mixtures were filtered to separate the solid phase. The C_e_ (mg/L) of MET in the supernatant was determined at 232 nm using the calibration curve previously presented, and the obtained values were used to assess the influence of pH on adsorption performance. The Q_e_ (mg/g) was subsequently calculated using Equation (2).

### 2.10. Procedure for Testing the Influence of Initial Concentration

The influence of the initial MET concentration on the adsorption onto X7 was investigated in batch mode. A series of MET solutions with different initial concentrations—30, 50, 100, 150, 200, 250, 300, 400, 450, and 500 mg/L—were prepared while keeping the solution V (L) constant at 0.01 L. For each concentration, 0.05 g of X7 resin was added to the MET solution in 100 mL Erlenmeyer flasks. The suspensions were agitated at 175 rpm for a contact time corresponding to adsorption equilibrium (80 min) at T= 25 ± 2 °C. After equilibration, the solid and liquid phases were separated by filtration, and the equilibrium concentration of MET in the supernatant was determined at 232 nm using the calibration curve. The adsorption capacity Q_e_ and the removal efficiency R (%) were subsequently calculated using Equations (2) and (3).

### 2.11. Procedure for Studying the Influence of Desorption Agents

A desorption study was carried out to evaluate the efficiency of various desorbing agents in regenerating the X7 resin previously saturated with MET. The X7-MET loaded with 62 mg/g was used as the material for all desorption experiments. Samples of 0.01 g of loaded resin were brought into contact with 20 mL of each desorbing solution under identical conditions. The desorption agents tested included 1 M HCl, 1 M NaOH, MeOH, EtOH, acetone, and AcCN, as well as 1:1 (*v*/*v*) mixtures of 1 M HCl with MeOH, EtOH, acetone, and AcCN, respectively. Each resin–solution mixture was agitated at 175 rpm for 30 min at T = 25 ± 2 °C to promote desorption. After agitation, the suspensions were filtered to separate the solid and liquid phases. The concentration of MET released into the supernatant was quantified spectrophotometrically, and the desorption efficiency of each agent was calculated. The desorption rate (D (%)) of MET from the X7–MET resin was calculated using a simplified expression that quantifies the fraction of MET released into the desorption medium relative to the amount initially retained by the polymeric matrix, Equation (4):

(4) $$D(\%)=\frac{A}{B}\times 100$$

where A represents the mass of MET (mg) released into the liquid phase during the desorption step; B corresponds to the mass of MET (mg) initially adsorbed onto the X7 resin prior to desorption.

## 3. Results

### 3.1. Influence of Initial pH on the Adsorption of MET onto X7

The adsorption capacity of MET on the nonionic polymeric resin X7 was evaluated in the pH range 2.0–10.2. The obtained adsorption capacities (36.23–36.39 mg/g) exhibit only minimal variation across the entire pH interval, indicating that the adsorption process is essentially pH-independent.

**Figure 2:**
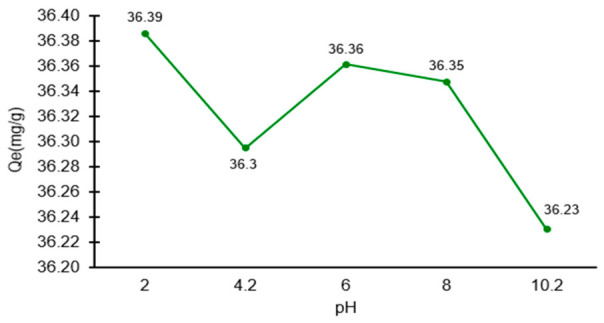
Effect of initial pH and buffer composition on MET adsorption onto X7. Data represent mean values of duplicate experiments with standard deviation below 3%.

Figure 2 shows the relationship between pH and equilibrium adsorption capacity (Q_e_), confirming the negligible influence of pH and buffer type on adsorption performance.

MET possesses two relevant pKa values (pKa_1_ ≈ 2.8 and pKa_2_ ≈ 11.5). Consequently, in the pH range investigated, the molecule exists exclusively in cationic forms: the doubly protonated species (H_2_MET^2+^) at pH < 2.8 and the monoprotonated species (HMET^+^) between pH 3 and 10. Since no neutral species is formed in this interval, the hydrophobicity of MET does not undergo significant changes with pH. Given that X7 is a nonionic, moderately polar acrylic resin, the adsorption mechanism is dominated by hydrophobic interactions, dipole–dipole forces and hydrogen bonding. This explains the remarkable stability of Q_e_ values despite changes in pH and buffer composition.

In addition, the buffer systems employed—phosphate (pH 2.0 and 8.0), acetate (pH 4.2 and 6.0) and carbonate (pH 10.2)—may influence the ionic strength and hydrogen-bonding environment of the solution.

At pH 2.0, MET is predominantly in its doubly protonated form (H_2_Met^2+^). Although the molecule carries a higher positive charge, its relative hydrophobicity increases slightly due to the compact structure of the guanidinium moiety. This favors its interaction with the moderately hydrophobic matrix of X7. The highest adsorption capacity in the series (36.39 mg/g) is recorded at this pH, though the difference is marginal. The phosphate buffer does not interfere with adsorption, as phosphate ions do not compete for binding sites on the nonionic resin.

At pH 4.2, MET exists exclusively as HMET^+^, a highly soluble monoprotonated species. The acetate buffer provides a mildly polar environment, but acetate ions do not significantly compete with MET for interactions with the resin. The adsorption capacity (36.30 mg/g) remains nearly identical to that at pH 2.0, confirming that the transition from H_2_Met^2+^ to HMET^+^ does not alter the adsorption mechanism.

The same monoprotonated species dominates at pH 6.0. The adsorption capacity (36.36 mg/g) is virtually unchanged, indicating that neither the degree of protonation nor the acetate buffer composition affects the interaction between MET and X7. This stability highlights the hydrophobic-driven nature of the adsorption process.

At pH 8.0, MET remains monoprotonated. Although phosphate ions can increase the ionic strength of the solution, no competitive adsorption is observed. The Q_e_ value (36.35 mg/g) confirms that the resin’s adsorption performance is unaffected by the presence of multivalent anions or by moderate alkalinity.

At pH 10.2, the system approaches the second pKa of MET (11.5), but the molecule remains predominantly monoprotonated. Carbonate and bicarbonate ions from buffer solution may slightly disrupt hydrogen-bonding networks in solution and increase MET solubility, which could explain the minor decrease in Q_e_ (36.23 mg/g). Nevertheless, the variation is negligible, reinforcing the conclusion that adsorption is not governed by electrostatic interactions. The pH independence observed for X7 is consistent with the literature on the adsorption of polar compounds onto nonionic acrylic resins [53,54,55,56,57,58]. The speciation of metformin in the pH range 2–10 is dominated by stable cationic forms [29,37,38,39,40,42], which explains the absence of significant variations in hydrophobicity and, consequently, in adsorption capacity.

### 3.2. Effect of Adsorbent Dosage on MET Adsorption onto X7

**Figure 3:**
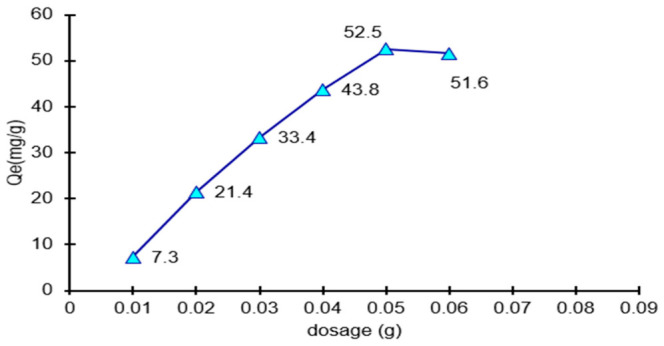
Influence of adsorbent dosage on MET adsorption onto X7. Data represent mean values of duplicate experiments with standard deviation below 3%.

The influence of adsorbent dosage on the adsorption capacity of MET was evaluated in the range 0.01–0.06 g at a fixed initial concentration (C_i_ = 300 mg L^−1^) and constant V = 0.01 L. The results illustrated in Figure 3 reveal a clear dosage-dependent evolution of the equilibrium adsorption capacity (Q_e_), reflecting the interplay between available binding sites, surface saturation and solute distribution in the liquid–solid system [70].

At low dosage (0.01 g), the system exhibits a modest Q_e_ of 7.3 mg/g, attributable to the limited number of accessible adsorption sites relative to the high solute load. As the dosage increases to 0.02–0.04 g, Q_e_ rises sharply (21.4–43.8 mg/g), indicating that the progressive introduction of additional active sites enhances MET uptake efficiency. This region corresponds to the site-controlled regime, where the number of available functional groups on X7 is the dominant factor governing adsorption.

A further increase to 0.05 g results in the maximum Q_e_ value (52.5 mg/g), as shown in Figure 3, reflecting an optimal balance between adsorbent surface area and solute availability. At this dosage, the resin provides sufficient accessible sites to accommodate MET molecules without significant competition or steric hindrance, while the solute concentration remains high enough to drive efficient site occupation.

Beyond this optimum, a slight decrease in Q_e_ is observed at 0.06 g (51.6 mg/g). This decline, also visible in Figure 3, is characteristic of the solute-limited regime, where the total amount of MET in solution becomes insufficient to fully utilize the increasing number of adsorption sites. As a result, the adsorption capacity per unit mass decreases, even though the total amount of MET removed from solution may remain constant or increase marginally.

Overall, the dosage study shows that MET adsorption onto X7 is primarily governed by the balance between available binding sites and solute concentration. Maximum adsorption efficiency is reached at 0.05 g, beyond which Q_e_ stabilizes or slightly decreases due to underutilization of the adsorbent surface. These results confirm that the process is dictated by surface accessibility and solute distribution, consistent with the nonionic nature of X7 and the interaction mechanisms previously described. Also, the dosage-dependent evolution of Q_e_ reflects classical site-controlled and solute-limited adsorption behavior, consistent with the literature on polymeric adsorbents [53,54,55,56,57,58].

### 3.3. Effect of Contact Time on the Adsorption of MET onto X7

Contact time between the liquid phase and the adsorbent surface is a key operational parameter in adsorption processes, as it governs both the rate of solute uptake and the time required to reach equilibrium. According to the adsorption literature, the temporal evolution of the process is typically controlled by mass-transfer phenomena and by the accessibility of the adsorbent’s porous structure, with an initial rapid uptake followed by a slower stage associated with intraparticle diffusion [71,72,73].

**Figure 4:**
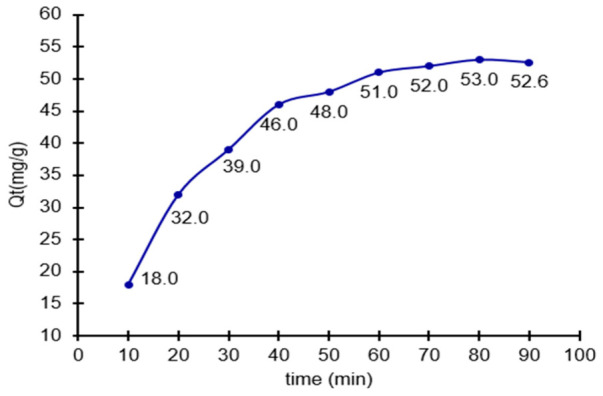
Influence of contact time on MET adsorption onto X7. Data represent mean values of duplicate experiments with standard deviation below 3%.

For the X7-MET system, the effect of contact time was investigated at the optimal initial concentration (C_i_ = 300 mg/L) and an adsorbent mass of 0.05 g. The experimental results are presented in Figure 4.

A pronounced increase in Q_e_ is observed during the first 40–50 min, where the adsorption process is dominated by external mass transfer toward the resin surface. In this interval, the adsorption capacity rises from 18.0 mg/g (10 min) to 48.0 mg/g (50 min), corresponding to an increase in removal efficiency from 30% to 80%. Such behavior is characteristic of macroporous polymeric adsorbents, where the readily accessible external surface is rapidly occupied during the early stages of the process.

Between 50 and 80 min, the increase in Q_e_ becomes gradual, reaching 52–53 mg/g. This transition marks the onset of the intraparticle diffusion regime, during which MET molecules progressively penetrate the internal domains of the polymer matrix. The slower uptake reflects the progressive saturation of accessible sites and the reorganization of interfacial interactions within the porous network.

After approximately 80–90 min, the system reaches adsorption equilibrium, with Q_e_ stabilizing at ~52.6–53.0 mg/g and the removal efficiency approaching ~88%. The minor variations in Ce observed in this interval confirm that the active surface of the resin is nearly saturated and that further uptake is limited by the decreasing availability of MET molecules in solution.

The observed behavior is consistent with the adsorption mechanism previously proposed for this system, in which directional N–H···O=C and C=N···C=O interactions, together with dipolar coupling, contribute to the stabilization of MET at the polymer interface. The progressive establishment of these interactions explains the shift from the rapid initial stage to the slower diffusion-controlled phase.

To conclude, the experimental data indicate that a contact time of ≈ 80 min is sufficient to achieve equilibrium in the MET–X7 system. The temporal evolution of Q_e_ confirms a two-stage adsorption mechanism: an initial fast uptake governed by external mass transfer, followed by a slower phase dominated by intraparticle diffusion and the organization of directional interfacial interactions.

### 3.4. Adsorption Kinetics

The adsorption kinetics of MET on X7 were evaluated using the intraparticle diffusion (Weber–Morris), Elovich, pseudo-first-order (PFO), and pseudo-second-order (PSO) models, whose linearized equations are presented in Table 1 and in Figure S1 in the Supplementary Materials [67,68,72,74]. The intraparticle diffusion model (Equation (5)) was used to assess whether the transport of MET molecules inside the polymeric matrix contributes significantly to the overall rate. A linear dependence of Qt on t^0.5^ would indicate diffusion-controlled uptake. The Elovich model (Equation (6)) was applied to analyze adsorption on heterogeneous surfaces, where the adsorption rate decreases progressively as surface sites become occupied. This model is suitable for polymeric adsorbents with a broad distribution of surface energies.

The PFO (Equation (7)) was tested to determine whether the adsorption rate is governed primarily by external mass transfer and weak surface interactions. The agreement between calculated and experimental $q_{e}$ values was used to evaluate the adequacy of this model.

The PSO (Equation (8)) was also examined, not to imply chemical bonding, but because PSO frequently provides an excellent empirical fit for systems where the adsorption rate is influenced by both surface interaction and diffusion processes. In polymeric resins such as X7, a good PSO fit typically reflects a combined contribution of film diffusion, intraparticle diffusion, and non-specific physical interactions, rather than a true chemical reaction at the surface. Comparison of the R^2^ obtained for all four models allowed identification of the dominant rate-controlling steps governing MET uptake on X7.

Weber–Morris (intraparticle diffusion):

(5) $$q_{t}=k_{id}{\left(t\right)}^{0.5}+C$$

Elovich:

(6) $$q_{t}=\ln(\alpha \beta )+\frac{1}{\beta }\mathrm{lnt}$$

PFO:

(7) $$\log\left(q_{e}-q_{t}\right)=\log q_{e}-\left(\frac{k_{1}}{2.303}\right)t$$

PSO:

(8) $$\frac{t}{q_{t}}=\frac{1}{k_{2}q_{e}^{2}}+\frac{t}{q_{e}}$$

The PSO model exhibited the highest correlation (R^2^ = 0.9881), indicating that this model provides the most accurate description of the adsorption kinetics. The excellent agreement between experimental and calculated equilibrium capacities (Q_e_, _exp_ = 78.7 mg/g) suggests that the rate-limiting step is associated with surface interactions and the availability of active sites, rather than mass-transfer limitations. Although PSO is often associated with physisorption, the low adsorption energies obtained from isotherm modeling (Temkin and D–R) confirm that the process remains physisorption-controlled, with PSO reflecting the kinetic form rather than a chemical mechanism.

The PFO model showed a lower correlation (R^2^ = 0.9259) and underestimated the equilibrium capacity (Q_e_ = 76.5 mg/g), indicating that the initial uptake is rapid but the model fails to capture the later stages of adsorption. This deviation confirms that MET adsorption does not follow a simple diffusion-controlled first-order process.

The Elovich model provided a strong fit (R^2^ = 0.9700), suggesting that the adsorption surface exhibits a degree of energetic heterogeneity. The relatively high initial adsorption rate (α = 1.91 mg/g·min) and low desorption constant (β = 0.06 g/mg) indicate fast initial uptake followed by progressive site saturation. This behavior is consistent with polymeric adsorbents where surface accessibility is high but gradually decreases as loading increases.

The Weber–Morris intraparticle diffusion model showed a moderate correlation (R^2^ = 0.8971) and a non-zero intercept (C = 7.43 mg/g), demonstrating that intraparticle diffusion contributes to the overall rate but is not the sole rate-limiting step. The intercept magnitude indicates a significant boundary-layer effect, confirming that external film diffusion governs the early stages of adsorption, followed by slower diffusion into the polymeric matrix.

Overall, the kinetic hierarchy is: PSO (0.9881) > Elovich (0.9700) > PFO (0.9259) > Weber–Morris (0.8971).

This ranking demonstrates that MET adsorption onto X7 is best described by a pseudo-second-order kinetic model, with a mechanism dominated by rapid surface uptake followed by slower intraparticle diffusion, fully consistent with the physisorption-driven monolayer behavior identified in the isotherm analysis.

### 3.5. Adsorption of MET onto X7 as Function of Initial Concentration

**Figure 5:**
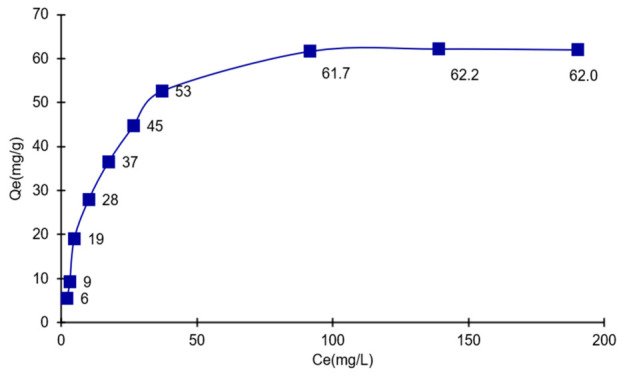
Equilibrium data for MET adsorption on X7 resin; data represent mean values of duplicate experiments with standard deviation below 3%.

The adsorption capacity of X7 toward MET was assessed across a broad range of initial concentrations (30–500 mg/L), under constant experimental conditions. The corresponding results are shown in Figure 5. At low C_i_ (30–100 mg/L), the resin exhibited high removal, ranging from 92.8% to 95.2%, corresponding to Q_e_ values between 6 and 19 mg/g. This performance reflects the strong affinity of MET for the moderately polar acrylic matrix of X7, where hydrogen bonding and electrostatic interactions dominate the uptake mechanism. The high efficiency in this region indicates that the available adsorption sites are abundant relative to the solute concentration, enabling nearly complete removal of MET from solution.

As the initial concentration increased from 150 to 300 mg/L, the Q_e_ continued to rise, reaching 28–53 mg/g, while the R (%) gradually decreased to 87–93%.

This trend is typical for polymeric adsorbents: although more MET is taken up per gram of resin, the proportion removed from solution decreases because the number of available active sites and surface area become progressively limited. The transition from near-complete removal to moderate removal efficiency marks the onset of site saturation.

At the highest concentrations tested (400–500 mg/L), the resin approached its saturation region. The adsorption capacity reached a plateau around 62 mg/g, while the removal efficiency dropped significantly to 77.1%, 69.1%, and 62.0%, respectively. This plateau behavior indicates that the majority of accessible adsorption sites were occupied, and additional increases in C_i_ no longer produced proportional increases in Q_e_.

Overall, the adsorption profile demonstrates that X7 possesses a high affinity and substantial adsorption capacity for MET, particularly at environmentally relevant concentrations (<100 mg/L). The X7 resin maintains strong performance up to moderate concentrations, after which site saturation becomes the limiting factor. These findings confirm that X7 is a promising adsorbent for MET removal from aqueous matrices, combining high efficiency at low concentrations with a relatively large maximum uptake capacity at equilibrium.

Based on the comparative adsorption data summarized in Table 2, the performance of Amberlite X7 can be positioned relative to the main classes of adsorbents previously reported for metformin removal. Amberlite X7 provides an optimal compromise between performance, cost, and regenerability, exhibiting an adsorption capacity (62 mg/g) higher than biochars (7.53–16.79 mg/g) [54], silica–alumina (45.7 mg/g) [44] and Fe ZSM 5 (14.99 mg/g) [32], comparable to activated carbons (50.99–122.47 mg/g) [59,75], and lower than graphene-based materials (90–232 mg/g) [30,31,43,53]. Its practical advantages—chemical stability, commercial availability, and efficient regeneration with minimal capacity loss—make it far more feasible for real-world applications than expensive or difficult-to-regenerate materials such as graphene oxide, MOFs, or metal oxide nanocomposites [30,31,43,53,76]. Overall, X7 offers the best combination of efficiency, cost, and reusability, fully justifying its selection as the adsorbent in this study.

#### Adsorption Isotherm Modeling

A quantitative assessment of adsorption equilibrium is essential for elucidating how MET interacts with the surface of the adsorbent. Adsorption isotherms define the relationship between the equilibrium adsorption capacity (Q_e_) and the solute concentration at equilibrium (C_e_), providing insight into surface characteristics, energetic heterogeneity, and the governing adsorption mechanism [77,78]. To describe the equilibrium uptake of MET on X7, the Langmuir (Equation (9)), Freundlich (Equation (10)), Temkin–Pyzhev (Equations (11) and (12)), and Dubinin–Radushkevich (Equations (13)–(15)) models were applied, and the results are presented in Table 3 and in Figure S2 in the Supplementary Materials [67,79,80,81].

(9) $$\frac{C_{e}}{Q_{e}}=\frac{1}{bQ_{m}}+\frac{C_{e}}{Q_{0}}$$

(10) $$\ln Q_{e}=\ln K_{f}+\frac{1}{n}{\mathrm{lnC}}_{e}$$

(11) $$Q_{e}=\frac{\mathrm{RT}}{b_{T}}\ln\;(AC_{e})$$

(12) $$\mathrm{and}\;\mathrm{can}\;\mathrm{be}\;\mathrm{linearized}\;\mathrm{as}:\;Q_{e}=\mathrm{BlnA}+\mathrm{Bln}C_{e}$$

(13) $$\ln Q_{e}=\ln q_{m}-\beta \varepsilon^{2}$$

(14) $$\varepsilon =\mathrm{RTln}(1+\frac{1}{C_{e}})$$

(15) $$E=\frac{1}{\sqrt{2\beta }}$$

The Langmuir model yielded the highest correlation (R^2^ = 0.9954), indicating an agreement with the experimental data and confirming that MET uptake proceeds predominantly through monolayer adsorption on a homogeneous surface. The monolayer capacity (Q_o_ = 68.5 mg/g) is consistent with the moderate hydrophilicity of X7 and the molecular size of MET, while the affinity constant (b = 0.063 L/mg) reflects a balanced interaction strength typical for polymeric adsorbents. The dimensionless separation factor (R_L_ = 0.05) lies well within the favorable adsorption region, reinforcing the suitability of the Langmuir model. Thus, these parameters indicate that the X7 surface offers energetically uniform sites and that the adsorption process is dominated by site-specific interactions rather than multilayer accumulation.

The Temkin–Pyzhev model exhibited a moderate fit (R^2^ = 0.8839), suggesting that although the heat of adsorption decreases with increasing surface coverage, this linear decay does not fully capture the experimental behavior. The Temkin binding energy (b_T_ = 181.3 J/mol) is relatively low, consistent with physisorption, and aligns with the weakly polar nature of the polymeric matrix. The Temkin constant (B = 13.7) further indicates a narrow distribution of adsorption energies, but it is insufficient to rival the uniformity implied by the Langmuir fit.

The Freundlich model produced the lowest correlation (R^2^ = 0.8549), indicating that surface heterogeneity plays a limited role in this system. Although the Freundlich constant (n = 2.01) confirms favorable adsorption (n > 1), the model’s empirical nature and weaker fit suggest that multilayer adsorption or highly heterogeneous surface energies are not dominant mechanisms. The Freundlich constant (K_f_ = 6.63 mg/g) reflects moderate adsorption intensity, consistent with the polymer’s nonionic character.

The D–R model provided a correlation comparable to Temkin (R^2^ = 0.8856), offering additional insight into the adsorption mechanism. The mean adsorption energy (E = 4.08 kJ/mol) is below the 8 kJ/mol threshold, unequivocally indicating physisorption, dominated by weak electrostatic or van der Waals interactions. The theoretical capacity (qm = 46.82 mg/g) is lower than the Langmuir monolayer capacity, suggesting that the D–R model underestimates the actual uptake, likely due to its assumption of Gaussian energy distribution, which is less appropriate for polymeric adsorbents.

Overall, the hierarchy of model performance is: Langmuir (0.9954) > D–R (0.8856) > Temkin (0.8839) > Freundlich (0.8549).

This ranking demonstrates that MET adsorption onto X7 is best described by a Langmuir-type monolayer mechanism, governed by physisorption and characterized by favorable uptake across the investigated concentration range. The combined evidence from Langmuir, Temkin, and D–R parameters converges toward a mechanism dominated by weak, non-specific interactions on a surface with relatively uniform energy distribution.

### 3.6. Desorption Studies

**Figure 6:**
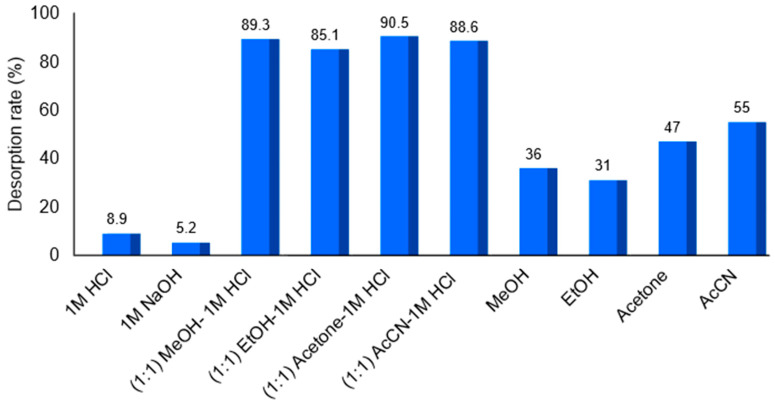
Desorption of MET from X7 using aqueous, organic, and mixed organic–acidic eluents; data represent mean values of duplicate experiments with standard deviation below 3%.

The desorption studies obtained with different eluents are summarized in Figure 6, which clearly illustrates the contrasting performance of aqueous, organic, and mixed solvent systems [82].

As shown in Figure 6, single-phase aqueous eluents exhibited very low desorption efficiencies, with only 8.9% MET removed using 1 M HCl and 5.2% using 1 M NaOH. These results are fully consistent with the adsorption mechanism previously established: MET interacts strongly with both polar and moderately hydrophobic domains of X7, forming a stable monolayer as predicted by the Langmuir model. Although MET is fully protonated in acidic media, its strong hydration shell and limited affinity for the aqueous phase prevent efficient displacement from the resin. Similarly, alkaline conditions do not weaken the adsorption forces identified in the kinetic analysis. Thus, aqueous media alone cannot reverse the adsorption equilibrium, confirming the robustness of the interactions described earlier.

Pure organic solvents significantly improved desorption relative to aqueous eluents, following the order illustrated in Figure 6: EtOH (31%) < MeOH (36%) < acetone (47%) < acetonitrile (55%).

This trend mirrors the polarity-dependent adsorption behavior inferred from the isotherm analysis. Acetonitrile, the most polar aprotic solvent tested, achieved the highest desorption (55%), suggesting that efficient solvation of MET and enhanced penetration into the macroreticular pore network are key factors. However, the incomplete desorption obtained with all pure organic solvents indicates that organic solvation alone cannot fully disrupt the adsorption interactions, which were shown to be strong and rapid in the kinetic section.

An increase in desorption efficiency was observed when organic solvents were combined with 1 M HCl in a 1:1 ratio. As depicted in Figure 6, all mixed eluents achieved desorption above 85%, demonstrating a strong synergistic effect between protonation and organic solvation: (1:1) acetone–1M HCl: 90.5%; (1:1) MeOH–1M HCl: 89.3%; (1:1) AcCN–1M HCl: 88.6%; and EtOH–HCl (1:1): 85.1%. These results correlate directly with the adsorption mechanism proposed earlier. The Langmuir-type monolayer adsorption and the PSO-controlled kinetics suggest strong surface interactions that require simultaneous disruption of electrostatic, dispersive, and solvation forces. Mixed organic–acidic systems achieve this through: (i) complete protonation of MET, reducing its affinity for the hydrophobic domains identified in the isotherm analysis; (ii) enhanced solvation in the organic phase, which stabilizes MET in solution and shifts the adsorption–desorption equilibrium; and (iii) improved pore penetration, consistent with the rapid intraparticle diffusion observed in the kinetic profile.

The near-complete desorption (>88%) confirms that regeneration efficiency is maximized when both chemical (acidic) and physical (organic solvent) mechanisms act simultaneously, fully reversing the adsorption pathways described in the previous sections.

From a strictly performance-based perspective, (1:1) acetone–1 M HCl is the most effective eluate, achieving 90.5% desorption and ensuring near-complete regeneration of the resin. However, considering operational aspects such as solvent cost, toxicity, ease of handling, and compatibility with repeated regeneration cycles, (1:1) MeOH–1 M HCl emerges as the most balanced option. With a desorption efficiency of 89.3%, it offers performance comparable to acetone–HCl while providing superior practicality for routine use.

The desorption results reinforce the conclusions drawn from adsorption, kinetic, and isotherm studies: MET exhibits strong, rapid, and energetically uniform adsorption onto X7, requiring a mixed organic–acidic system for efficient removal. The regeneration behavior is fully consistent with the mechanistic insights obtained earlier, confirming the suitability of X7 for repeated use in pharmaceutical removal applications.

### 3.7. FTIR Analysis

#### 3.7.1. FTIR Spectral Characterization

FTIR spectroscopy is a well-established analytical technique for the characterization of intermolecular interactions in drug–adsorbent systems [83]. By comparing the vibrational spectra of individual components with that of the resulting matrix, it is possible to identify spectral shifts, band disappearances, and intensity changes that are diagnostic of specific interaction mechanisms, including hydrogen bonding, electrostatic interactions, and van der Waals forces. The present study reports the comparative FTIR spectroscopic analysis of X7, MET, and MET–X7, with the aim of characterizing the adsorption mechanism and assessing the efficiency of drug uptake.

#### 3.7.2. FTIR Analysis of X7

**Figure 7:**
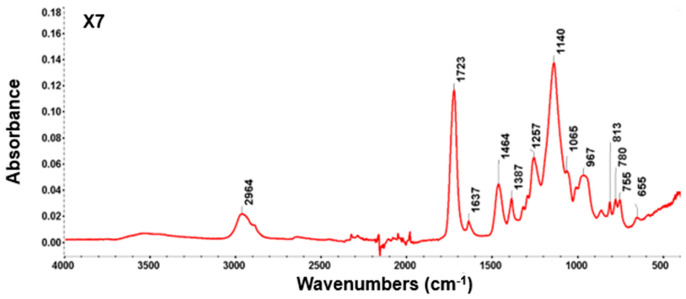
FTIR spectrum of X7 resin.

Figure 7 displays a set of characteristic absorption bands consistent with the acrylic ester polymer backbone of the resin.

The band at 2964 cm^−1^ is assigned to the asymmetric C–H stretching vibrations of aliphatic methylene (–CH_2_–) and methyl (–CH_3_) groups constituting the polystyrene-based skeletal framework. The medium intensity of this band is consistent with the predominantly apolar hydrocarbon character of the matrix.

A prominent absorption band at 1723 cm^−1^ is attributed to the C=O stretching vibration of ester functional groups, confirming the acrylic ester nature of Amberlite X7. This band is a key diagnostic marker for monitoring interactions with adsorbate molecules capable of hydrogen bond donation.

The band at 1637 cm^−1^ is of weak-to-moderate intensity and is tentatively attributed to residual C=C aromatic stretching or to adsorbed water deformation modes. Bands at 1463 and 1387 cm^−1^ correspond to C–H bending vibrations of –CH_2_– and –CH_3_ groups, respectively, reflecting the aliphatic character of the polymer chain.

The absorption at 1256 cm^−1^ is assigned to the asymmetric C–O–C stretching mode of the ester linkage, while the dominant band at 1140 cm^−1^ corresponds to the symmetric C–O–C stretching of the ester group. The latter represents the most intense and structurally diagnostic band of Amberlite X7, serving as an internal reference for monitoring the integrity of the resin upon adsorption.

The fingerprint region (400–1000 cm^−1^) displays multiple out-of-plane C–H deformation bands at 967, 812, 779, and 754 cm^−1^, consistent with aromatic ring substitution patterns of the polystyrene skeleton.

#### 3.7.3. FTIR Analysis of MET

**Figure 8:**
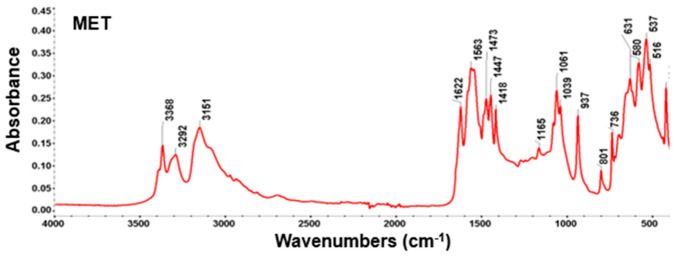
FTIR spectrum of MET.

Figure 8 is dominated by the characteristic vibrational profile of the biguanide functional group and the protonated amine moieties of the hydrochloride salt.

The broad band of the MET absorption envelope in the 3000–3500 cm^−1^ region encompasses three resolved bands: a sharp band at 3367 cm^−1^ (asymmetric N–H stretching of the primary amine –NH_2_), a shoulder at 3292 cm^−1^ (symmetric N–H stretching), and an intense, broad band at 3151 cm^−1^ attributed to N–H stretching in hydrogen-bonded aggregates or ionic N–H···Cl^−^ interactions characteristic of the hydrochloride salt form.

The multiplicity and breadth of these bands reflect the extensive hydrogen-bonding network in solid MET.

The region 1550–1650 cm^−1^ contains the most diagnostically significant bands for MET: the absorption at 1622 cm^−1^ is assigned to C=N stretching coupled with N–H in-plane bending of the biguanide system, while the intense band at 1563 cm^−1^ corresponds to asymmetric C–N stretching and N–H deformation modes. These bands represent the spectroscopic fingerprint of the biguanide moiety and are sensitive indicators of intermolecular interactions [26,27,83].

Additional bands at 1473, 1446, and 1417 cm^−1^ are attributed to C–N stretching and N–H deformation vibrations within the guanidinium framework. The bands at 1165, 1060, and 1039 cm^−1^ correspond to C–N stretching modes of the biguanide skeleton. The absorption at 937 cm^−1^ is consistent with out-of-plane N–H deformation of protonated amine species, corroborating the hydrochloride salt nature of the sample. The low-wavenumber region (400–800 cm^−1^) exhibits multiple bands at 800, 736, 631, 580, 536, and 515 cm^−1^, which are assigned to skeletal torsional and deformation modes specific to the biguanide framework.

#### 3.7.4. FTIR Analysis of X7–MET

**Figure 9:**
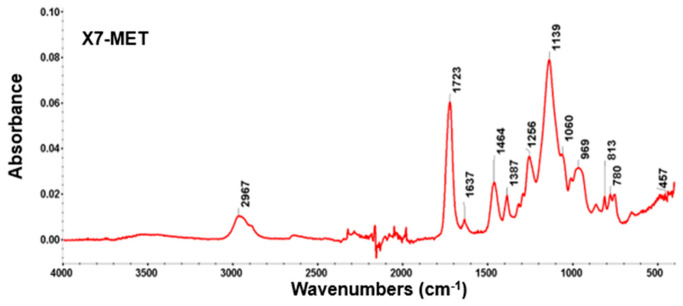
FTIR spectrum of the Amberlite X7–MET.

Figure 9 presents a spectral profile that cannot be interpreted as a simple superposition of the individual component spectra, providing direct spectroscopic evidence for intermolecular interactions between the drug and the resin.

The most significant observation is the complete disappearance of the N–H stretching bands of MET (3367, 3292, and 3151 cm^−1^) in the X7–MET spectrum. The absence—rather than a mere shift—of these bands in the adsorbed state strongly suggests that the N–H groups of MET are extensively engaged in hydrogen-bonding interactions with the acceptor sites of the resin, leading to a perturbation of the N–H oscillators sufficient to abolish their discrete absorption features.

The C=O stretching band of the Amberlite ester groups, located at 1723.23 cm^−1^ in the pure resin, is retained at 1723.17 cm^−1^ in the X7–MET, with a negligible wavenumber shift of approximately 0.06 cm^−1^. However, a reduction in relative intensity is observed with respect to the dominant C–O–C band at 1139 cm^−1^. This behavior is consistent with the involvement of the C=O group as a hydrogen bond acceptor in N–H···O=C interactions, wherein partial electron density delocalization weakens the carbonyl oscillator without producing a large wavenumber displacement.

The spectral region 1550–1640 cm^−1^, which is dominated by the biguanide C=N and N–H bending bands in pure MET (1622 and 1563 cm^−1^), shows only a weak broad absorption at approximately 1636 cm^−1^ in the X7–MET spectrum, attributable to the resin. The characteristic MET bands in this region are absent, indicating that the biguanide nitrogen atoms are directly involved in the drug–resin interaction, with the C=N and N–H vibrational modes being effectively quenched upon adsorption.

The C–H aliphatic stretching band of the resin undergoes a minor shift from 2964.31 to 2967.42 cm^−1^ in the X7–MET. While small, this shift may reflect subtle conformational changes in the polymer chains adjacent to adsorption sites or weak hydrophobic contributions to the overall interaction.

Crucially, the dominant C–O–C symmetric stretching band of Amberlite X7 is preserved at 1139.24 cm^−1^ in the X7–MET (compared to 1140.00 cm^−1^ in the pure resin), with no significant frequency shift or intensity loss. The conservation of this structurally diagnostic band confirms that the polymer backbone has undergone no covalent modification during the adsorption process, a finding consistent with physical adsorption.

#### 3.7.5. Nature of the Adsorption Mechanism

The collective FTIR evidence points to a physisorption mechanism involving two primary non-covalent interaction types.

The dominant interaction is hydrogen bonding of the N–H···O=C type, whereby the multiple N–H donor groups of MET’s biguanide moiety form hydrogen bonds with the ester carbonyl acceptor groups of Amberlite X7.

This interpretation is supported by: (i) the complete disappearance of all N–H stretching bands of MET; (ii) the suppression of the biguanide C=N/N–H bending modes; and (iii) the slight modification in relative intensity of the C=O band of the resin.

A secondary contribution from electrostatic and dipole–dipole interactions is also likely.

At physiological pH values, the biguanide moiety of MET (pKa ≈ 12.4) can adopt a protonated cationic form, which may interact electrostatically with the polar ester groups and the overall dipolar character of the Amberlite matrix [84,85,86]. Such electrostatic contributions would reinforce the hydrogen bonding and account for the extensive perturbation of the biguanide spectral features.

#### 3.7.6. Structural Integrity of the Adsorbent

The preservation of the C–O–C band at 1139 cm^−1^ and the C=O band at 1723 cm^−1^ (without significant frequency shifts indicative of ester hydrolysis or transesterification) confirms that the chemical structure of Amberlite X7 remains intact after MET loading [67,83]. No new bands attributable to covalent reaction products were detected in the X7–MET spectrum. These findings rule out physisorption and confirm that the drug–resin interaction is entirely physical in nature.

#### 3.7.7. Adsorption Efficiency

Comparative analysis: Figure 9 vs. Figure 7 and Figure 8.

##### Area 3000–3500 cm^−1^ (N–H)

This is the most informative spectral region for confirming adsorption. Pure MET (Figure 2/Spectrum 2) displays three well-resolved bands at 3367, 3292, and 3151 cm^−1^, with absorbance values of 0.15–0.19—the unambiguous vibrational signature of the –NH_2_ and =NH groups of the biguanide moiety. In the X7–MET spectrum (Figure 9/Spectrum 3), these bands are completely absent. They are not shifted but eliminated, indicating that the N–H groups of MET are strongly engaged in hydrogen-bonding interactions with the resin surface. A simple mechanical mixture without interaction would have retained these bands.

##### Area 1550–1640 cm^−1^ (C=N/δ N–H Biguanide)

Pure MET dominates this region with bands at 1622 and 1563 cm^−1^, with absorbance values of 0.26–0.32—the most intense features in its spectrum. In the X7–MET spectrum, these bands virtually disappear. Only a weak shoulder remains at approximately 1636 cm^−1^, attributable to the resin alone. The disappearance of the biguanide bands confirms that the C=N and N–H sites of MET are directly involved in the interaction with the polymer matrix.

##### C=O Band at ~1723 cm^−1^ (Amberlite X7)

Figure 7/Spectrum 1 presents this band at 1723.23 cm^−1^. In the X7–MET spectrum it appears at 1723.17 cm^−1^—essentially the same position—but with a reduced relative intensity compared to the dominant C–O–C band at ~1139 cm^−1^. This relative reduction, rather than a significant wavenumber displacement, indicates that the C=O groups act as hydrogen bond acceptors toward the N–H groups of MET, with partial electron density delocalization weakening the carbonyl oscillator without producing a large frequency shift.

##### C–O–C Band at ~1139–1140 cm^−1^ (Amberlite X7)

This is the dominant structural band of Amberlite X7. It appears at 1140.00 cm^−1^ in the pure resin spectrum and at 1139.24 cm^−1^ in the X7–MET—a difference of less than 1 cm^−1^.

The near-perfect conservation of this band is direct evidence that adsorption is physical in nature: the polymer chain of the resin has undergone no chemical transformation. This is an essential condition for adsorbent recyclability.

The efficiency of MET adsorption onto Amberlite X7 is substantiated by the following spectroscopic criteria:

Completeness of N–H band disappearance: all three N–H stretching bands of MET (3367, 3292, 3151 cm^−1^) are absent in the adsorption spectrum, indicating that a substantial fraction of the drug’s amino and imino groups are engaged in interactions with the resin surface. In a partial or low-efficiency adsorption scenario, residual unbound N–H bands would be expected.

Suppression of biguanide signature bands: the biguanide C=N and N–H bands at 1622 and 1563 cm^−1^, which are the most intense features in the MET spectrum, are effectively abolished in the X7–MET, suggesting high surface coverage and intimate contact between the drug molecules and the resin matrix.

Absence of free drug signature: the overall spectral profile of the X7–MET closely resembles that of pure X7, with no features identifiable as free (non-interacting) MET, which would indicate that essentially all spectroscopically detectable MET is in an adsorbed state.

#### 3.7.8. Adsorption of MET on X7 Confirmed by FTIR Analysis

**Figure 10:**
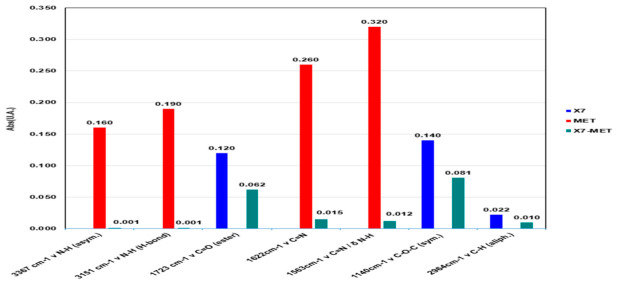
Comparative FTIR absorbance values for X7, MET, and the X7–MET at selected diagnostic bands.

The FTIR spectra of Amberlite X7, pure MET, and the X7-MET provide (Figure 10) direct spectroscopic evidence for the occurrence of adsorption, with the key band assignments summarized in Table 4.

The FTIR spectrum of the X7–MET shows the disappearance of five out of seven characteristic MET bands, including all N–H stretching vibrations (3367–3292 cm^−1^) and the C=N/N–H coupled modes of the biguanide group (1622–1563 cm^−1^), as detailed in Table 4. Their absence indicates that these functional groups are actively engaged in interactions with the ester groups of the resin. In a low-adsorption scenario, residual free N–H bands would remain detectable.

The spectral modifications reveal two concurrent interaction pathways: (a) N–H···O=C hydrogen bonding, supported by the disappearance of N–H bands and the slight decrease in intensity of the ester C=O band at 1723 cm^−1^ (Table 4); and (b) dipole–dipole or electrostatic interactions, consistent with the protonated biguanide moiety of MET and the polar acrylic ester surface of Amberlite X7. Together, these changes confirm that adsorption is not purely physical contact but involves directional, functional-group-specific interactions.

The selective involvement of N–H and C=N groups—while the aliphatic C–H region remains essentially unchanged (2964 → 2967 cm^−1^, Table 4)—demonstrates that adsorption is not governed by hydrophobic interactions. Instead, the resin exhibits specific affinity for the polar biguanide moiety, indicating a structurally selective adsorption mechanism.

The preservation of the characteristic C–O–C ester bands (1139 and 1256 cm^−1^ in Table 4) and the absence of any new absorption features confirm that no covalent bonds are formed during adsorption. This indicates a physisorption-dominated process, compatible with reversible desorption through pH or solvent polarity adjustments. The structural stability of the resin further supports its suitability for repeated adsorption–desorption cycles.

The combination of strong functional-group interactions, absence of chemical modification of the resin, and reversibility of the process positions the Amberlite X7/MET system as a promising candidate for pharmaceutical effluent treatment and potentially for controlled drug release applications. The FTIR evidence summarized in Table 4 provides a robust confirmation of the adsorption mechanism.

The disappearance of MET N–H and C=N bands in the X7–MET spectrum confirms adsorption onto the resin surface.

### 3.8. SEM Analysis

**Figure 11:**
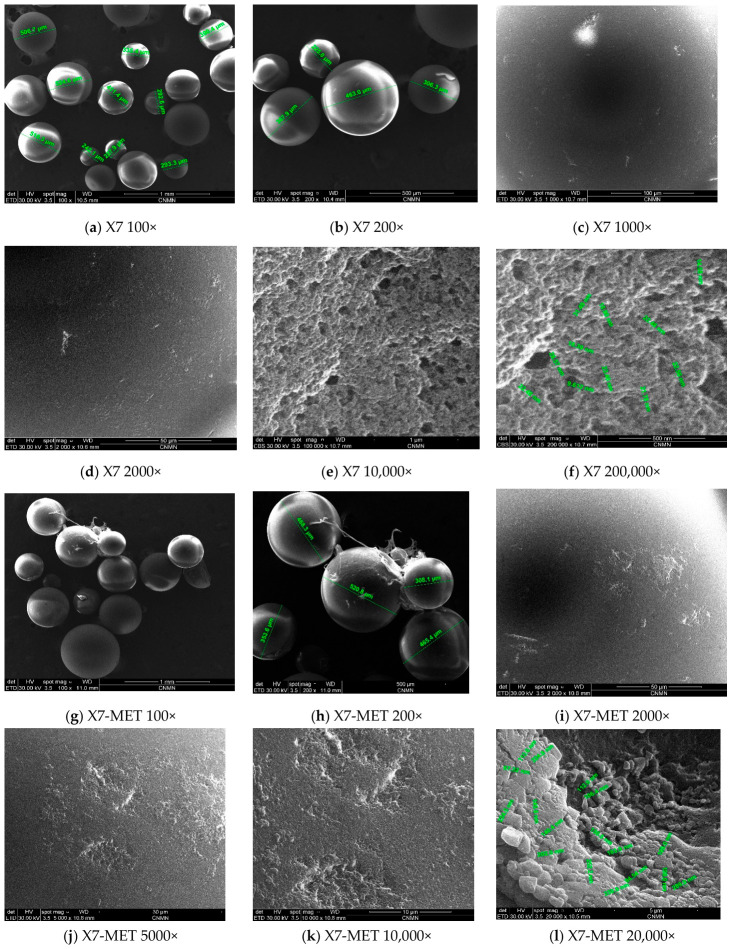
Surface and pore-scale SEM characterization of X7 during MET adsorption.

The SEM analysis provides a multiscale visualization of the morphological evolution of X7 before and after MET adsorption. The micrographs present in Figure 11a–f illustrate the structural characteristics of the X7 resin, while Figure 11g–l capture the progressive morphological changes associated with MET loading [68].

At low magnification (Figure 11a,b), the resin X7 exhibits the expected spherical geometry and clean surface typical of macroreticular acrylic resins, confirming the structural integrity of the polymer [87,88,89]. With increasing magnification (Figure 11c,d), a well-developed pore architecture becomes evident, supporting efficient external mass transfer and intraparticle diffusion [90,91]. High-magnification images (Figure 11e,f) reveal nanoscale surface irregularities associated with functional groups capable of engaging in hydrogen bonding, dipole–dipole alignment, and donor–acceptor interactions [92].

After MET adsorption, the X7–MET micrographs (Figure 11g–l) show a clear and systematic transformation of the surface. At low magnification (Figure 11g,h), contrast variations indicate the onset of surface deposition [93]. Intermediate magnification (Figure 11i,j) reveals granular material and partial pore coverage, confirming that adsorption involves both surface interaction and penetration into the pore network [94,95]. At the highest magnifications (Figure 11k,l), the adsorbed phase forms a continuous, compact nanoscale layer that masks the native roughness of the resin, indicating intimate interfacial organization and strong stabilization of the adsorbed MET layer [96].

Overall, the SEM evidence across Figure 11a–l demonstrates that MET adsorption on X7 is not superficial but involves surface coverage, pore-level deposition, and nanoscale consolidation, consistent with the cooperative adsorption mechanism inferred from FTIR, EDX, and kinetic analyses.

### 3.9. EDX Analysis

**Figure 12:**
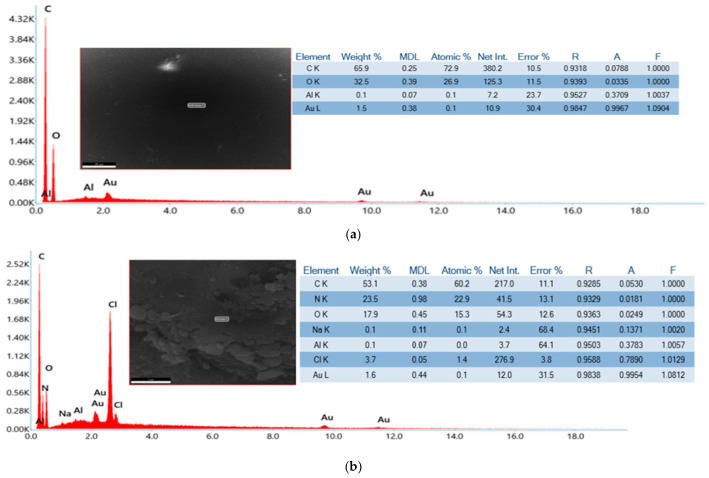

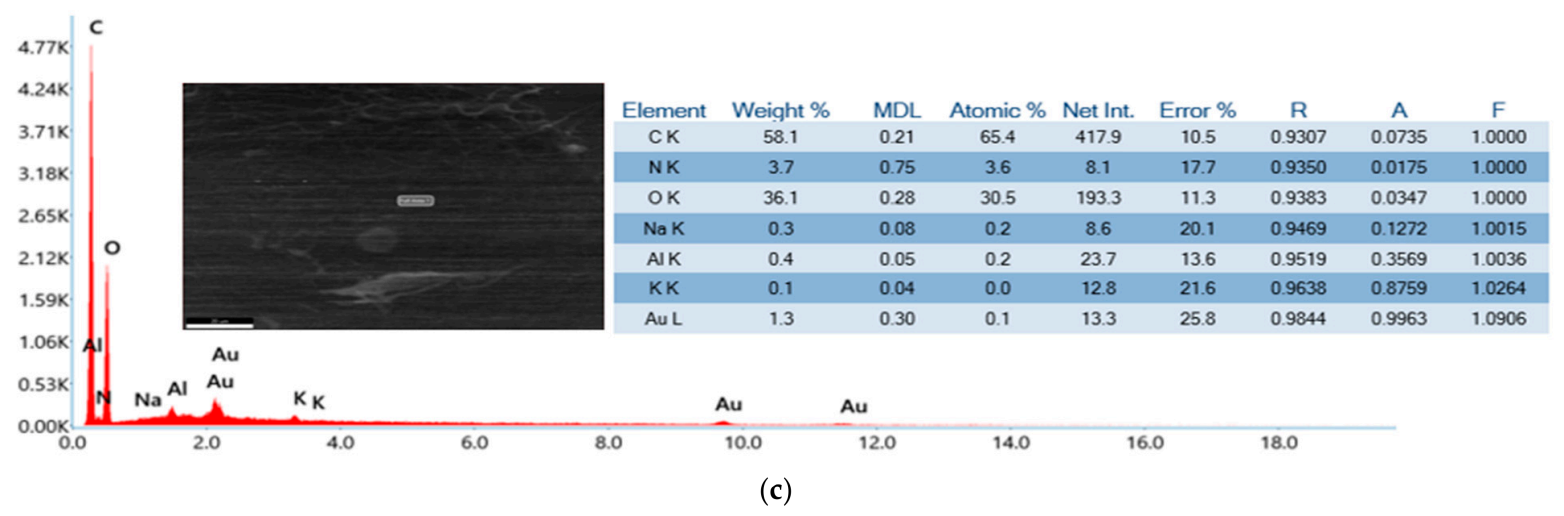
(**a**) EDS spectrum and elemental composition of X7; (**b**) EDS spectrum and elemental composition of MET; (**c**) EDS spectrum and elemental composition of X7-MET.

The EDX spectra shown in Figure 12a–c provide direct elemental evidence for MET adsorption onto X7 and clarify the nature of the interfacial interactions [68,97].

In Figure 12a, X7 resin exhibits the expected C/O profile characteristic of the acrylic ester matrix, with no detectable N or Cl. The minor Na, Al, K, and Au signals arise from trace impurities, substrate contributions, and gold sputtering during SEM preparation, and do not participate in adsorption. The spectrum of pure MET in Figure 12b is dominated by a strong N peak, originating from the biguanide core, and a pronounced Cl peak, corresponding to the chloride counterion. These elements serve as reliable markers for identifying MET in loaded material. After adsorption, Figure 12c shows the clear appearance of N in the X7–MET composite, unequivocally confirming MET retention on the resin surface. The increase in O intensity reflects the contribution of MET functional groups and supports the formation of N–H···O=C hydrogen bonds between MET and the ester carbonyls of X7. In contrast, the Cl peak is strongly attenuated, indicating that chloride is not co-retained and that adsorption proceeds through the neutral biguanide moiety. The persistence of Na, Al, K, and Au at similar trace levels across all samples confirms that no ion exchange or structural alteration of the resin occurs during adsorption. Instead, the spectral evolution (appearance of N, increase in O, disappearance of Cl) demonstrates the formation of an oriented C–O–N interfacial layer, consistent with directional N–H···O=C and C=N···C=O dipolar interactions. Overall, the EDX results corroborate the FTIR and SEM findings, confirming a selective, non-destructive adsorption mechanism in which MET binds through its biguanide core. At the same time, the polymer backbone remains structurally intact.

### 3.10. TG/DSC Analysis

**Figure 13:**
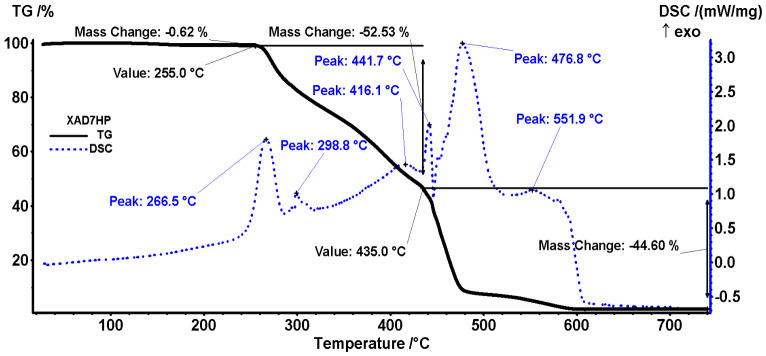
TG–DSC thermogram highlighting the mass loss stages and thermal transitions of the X7 sample.

The X7 is an acrylic cross-linked aliphatic resin with remarkable thermal stability up to 255 °C, with its recorded mass loss being 0.62%, most probably due to residual humidity from the water molecules trapped in the macropores [98]. The oxidative degradation onset is at 264.3 °C, close to previous reported values [72] (Figure 13).

The process is accompanied by a strong exothermic effect on the DSC curve, with the maximum at 266.5 °C generated by the oxidation of the branched aliphatic moieties. After this rapid oxidation, a slower mass loss occurs up to 435 °C, which generates multiple smaller exothermic peaks at 298.8 and 419.1 °C, due to fragmentation and oxidation reactions [72]. After 435 °C the degradation speed increases as oxidation processes complete, with the associated exothermic effect peaking at 476.8 °C. The residual carbonaceous mass is burned away after 500 °C, as indicated by the broad exothermic effect from 551.9 °C [99].

**Figure 14:**
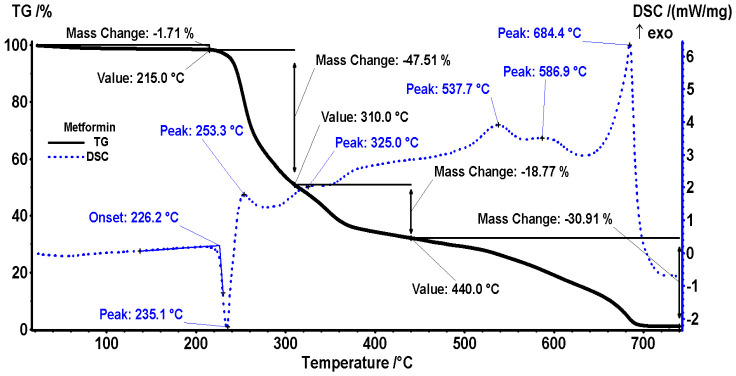
TG–DSC thermogram highlighting the mass loss stages and thermal transitions of the MET sample.

The MET sample also exhibits very good thermal stability up to 215 °C, with the recorded mass loss of 1.71% representing residual humidity from the drug (Figure 14). The sharp endothermic peak at 235.1 °C is characteristic for a melting process, but the TG curve also indicates an associated small mass loss, indicating the start of the thermal degradation. The melting onset is at 226.2 °C, with an area of 209.1 J/g, representing the melting enthalpy. These values are in good agreement with the previous literature reports [100,101,102].

The degradation of the drug occurs most probably by a complex process, in which fragmentation reactions are overlapped with oxidation reactions for the resulting fragments. The mass loss between 215 and 310 °C represents 47.51%, with the sinusoidal DSC shape confirming the complexity of the degradation, but with predominance of exothermic effects at 253.3 °C. In the temperature interval 310–440 °C the sample continues to lose mass, 18.77%, with the associated diffuse exothermic effect indicating oxidation as the principal degradation mechanism. After 440 °C the residual carbonaceous mass is burned away in a series of exothermic reactions, with the most intense effect at 684.4 °C. A similar behavior at high temperatures was reported in [103].

**Figure 15:**
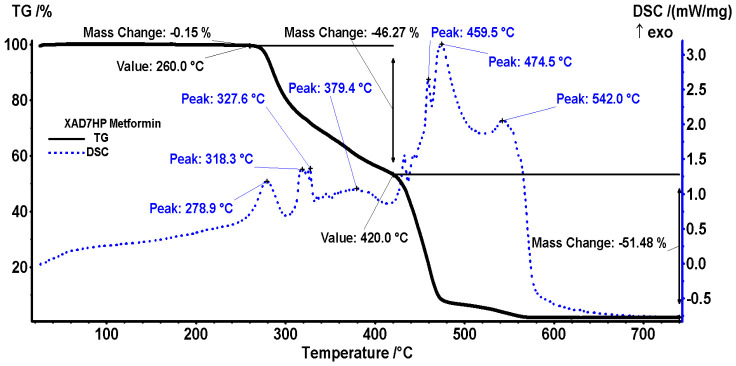
TG–DSC thermogram highlighting the mass loss stages and thermal transitions of the X7- MET sample.

The sample with MET adsorbed on XAD 7HP resin presents the best stability among the investigated samples. This behavior can be attributed to the interactions between MET and resin. When the MET is adsorbed on the Amberlite surface and into the pores, it creates a protective layer that slows the oxygen diffusion. At the same time the interactions between MET and XAD 7HP lead to blocking of the labile moieties, which improves the thermal stability. Thus, the sample loses only 0.15% up to 260 °C. The oxidative degradation is delayed by ~12 °C, as indicated by the maximum of the first exothermic peak, at 279.9 °C vs. 266.5 °C for XAD 7HP resin (Figure 15).

It is noteworthy that no melting endothermic peak can be observed for the sample XAD 7HP MET, indicating the disappearance of the crystalline structure for MET and its molecular entrapment mechanism [100], which is fundamentally different from the bulk loading process in nanostructured lipid carriers, for example [104].

The same shift towards higher temperatures can be observed for the next exothermic effect, which appears at 298.8 °C in the unloaded resin, but splits to 318.3 and 327.6 °C in the XAD 7HP MET sample. The splitting of the exothermic effect coupled with the increased mass loss speed after 300 °C indicates that the adsorbed MET starts to degrade. After 420 °C the principal mass loss step is recorded (51.48%), accompanied by multiple exothermic peaks on the DSC curve as the different residual carbonaceous masses (from the resin and from the drug) are burned away.

### 3.11. XRD Analysis

The MET exhibits a typical XRD pattern, with sharp, intense representative peaks, indicating a highly crystalline starting material [105,106].

**Figure 16:**
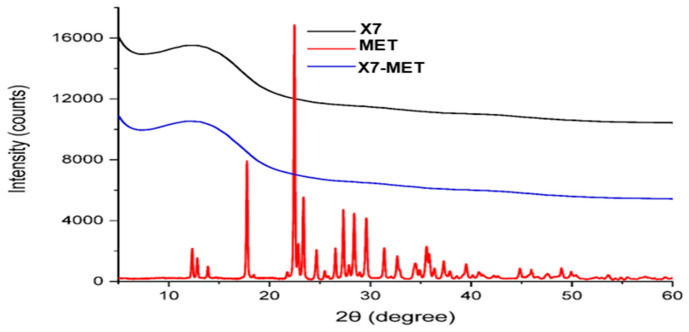
The XRD patterns for MET, X7 resin and X7 loaded with MET.

The most important peaks are at 2θ values 12.29°, 17.75°, 22.50°, 28.39°, 31.38° and 37.27°, corresponding to the crystalline pure MET (Figure 16) [107,108].

The absence of the characteristic MET peaks in the X7 sample loaded with MET indicates that MET lost its long-range structural order, with the molecules being in random positions. The broad halo peak from ~13.69° indicates an amorphous phase with the MET molecules being adsorbed individually on the resin’s surface, with no crystalline structure.

## 4. Discussion

The integrated analytical, structural, kinetic, and thermodynamic results reveal a coherent and reversible adsorption mechanism in which MET binds to the acrylic resin X7 through directional hydrogen bonding and dipolar interactions, as consistently supported by kinetic–isotherm modeling, FTIR, SEM/EDX, TG/DSC, and XRD, while the polymer matrix remains structurally intact.

The Q_e_ of X7 remains essentially constant across pH 2.0–10.2, confirming that MET uptake is pH-independent and governed by hydrogen bonding and dipolar interactions.

Kinetic modeling shows that the PSO model best describes the process, indicating that adsorption is controlled by active-site availability and directional interactions. The two-stage kinetic profile—rapid external mass transfer followed by slower intraparticle diffusion—is consistent with SEM evidence of both surface coverage and pore penetration.

Equilibrium data fit the Langmuir isotherm, confirming monolayer adsorption on uniform sites, with a high monolayer capacity (Q_0_ = 68.5 mg/g) and favorable separation factor. The low adsorption energy (E = 4.08 kJ/mol) indicates a physisorption-driven mechanism, while weaker fits of the Freundlich and Temkin models reinforce the predominance of site-specific monolayer adsorption.

Desorption studies show that acidic matrices are ineffective (<10%), while pure organic solvents provide moderate recovery (31–55%), and mixed organic–acidic systems enhance desorption (>85%), with acetone–1M HCl (1:1) reaching 90.5% and MeOH–1M HCl offering the best practical balance, confirming that X7 can be efficiently regenerated for circular water treatment applications.

FTIR spectroscopy provides the most direct evidence for the nature of the adsorption mechanism. The complete disappearance of the N–H stretching bands of MET (3367, 3292, 3151 cm^−1^) and the suppression of the biguanide C=N/N–H bending modes (1622, 1563 cm^−1^) in the X7–MET spectrum indicate that these functional groups are fully engaged in hydrogen bonding with the ester carbonyls of the resin. The minimal shift in the resin’s C=O band (1723 → 1723.17 cm^−1^) and the preservation of the C–O–C ester bands confirm that the polymer backbone undergoes no covalent modification, consistent with a physisorption mechanism. These findings align with the molecular-level model proposed earlier, where N–H···O=C hydrogen bonding, dipole–dipole alignment between C=N and C=O groups, and weak donor–acceptor orbital interactions collectively stabilize the adsorbed state.

SEM micrographs show that MET loading transforms the resin X7 surface from a rough, porous morphology into a smoother, partially occluded structure, with a continuous nanoscale layer masking the native pores. EDX analysis confirms MET adsorption through the clear appearance of N and the increase in O intensity, while the strong attenuation of Cl^−^ shows that binding occurs through the biguanide core, preserving the structural integrity of the X7 resin. TG/DSC analysis reveals enhanced thermal stability for X7–MET, with the first major exothermic peak shifting from 266.5 °C to 279.9 °C. The disappearance of the MET melting peak and the loss of crystalline reflections in XRD demonstrate molecular-level dispersion of MET within the polymer matrix, consistent with a stabilized and uniformly loaded resin. The combined results demonstrate that X7 is a highly effective and reusable adsorbent for MET, with performance governed by selective directional interactions, monolayer adsorption, molecular-level dispersion, and high regeneration efficiency, while its pH-independent behavior and structural stability further support its suitability for sustainable water treatment applications.

## 5. Conclusions

This study provides the first comprehensive investigation of MET adsorption onto the nonionic acrylic resin X7, integrating operational, mechanistic, structural, and regeneration analyses that were used to elucidate the suitability of this material for circular water treatment applications. The results demonstrate that X7 exhibits a remarkably stable adsorption performance across a wide pH range (2.0–10.2), reflecting the dominance of hydrogen bonding, dipole–dipole interactions, and donor–acceptor orbitals. The adsorption of MET onto X7 is remarkably stable over the pH range of 2–10, regardless of the buffer used. This stability is due to: (1) the permanently cationic nature of MET; (2) the absence of ionic groups on the X7 resin; and (3) the adsorption mechanism dominated by hydrophobic and dipole–dipole interactions.

The adsorption kinetics follow a PSO model, indicating physisorption-controlled uptake, while equilibrium data fit the Langmuir isotherm, confirming monolayer adsorption on a homogeneous distribution of active sites.

Although the adsorption experiments were performed in synthetic solutions, the practical relevance of the study is supported by the pH-independent adsorption behavior, the robustness of the Langmuir and pseudo-second-order fits, and the structural stability of XAD7HP under all tested conditions; nevertheless, we acknowledge that real wastewaters contain competing ions, natural organic matter and co-contaminants that may influence the performance of X7 resin.

The comparative FTIR spectroscopic analysis of X7, MET and their adsorption form has provided direct evidence for efficient physical adsorption of the drug onto the resin surface.

Spectra of the pure resin, MET, and the resulting X7-MET were recorded and systematically compared in the 400–4000 cm^−1^ range. The comparative spectral analysis revealed the disappearance of characteristic N–H stretching bands of MET (3367, 3292, and 3151 cm^−1^) and the attenuation of C=N/N–H bending vibrations (1622 and 1563 cm^−1^) in the X7-MET spectrum, indicating strong hydrogen-bonding interactions between the amino/imino groups of MET and the ester carbonyl groups of the resin. The structural integrity of X7, evidenced by the conservation of the C–O–C stretching band at 1139 cm^−1^, confirms physical adsorption. These aspects demonstrate a dual-mechanism adsorption process involving hydrogen bonding and dipole–dipole interactions, with implications for controlled drug delivery and pharmaceutical wastewater treatment.

The adsorption of MET on X7 proceeds via a physisorption mechanism, as demonstrated by the absence of new covalent bond formation and the complete conservation of the resin’s structural C–O–C band at ~1139 cm^−1^.

The primary adsorption interaction involves N–H···O=C hydrogen bonding between the amino and imino groups of the MET biguanide moiety and the ester carbonyl groups of the acrylic resin. This is evidenced by the complete disappearance of all N–H stretching bands (3367, 3292, 3151 cm^−1^) and the attenuation of biguanide C=N/N–H bending modes (1622, 1563 cm^−1^) in the X7-MET spectrum.

Secondary electrostatic and dipole–dipole interactions between the protonatable biguanide moiety of MET and the polar ester groups of the resin are likely to contribute to the overall binding affinity.

The spectroscopic evidence indicates good adsorption, as reflected by the complete suppression of free MET, N–H spectral features and the dominance of X7 characteristic bands in the X7-MET spectrum.

The reversibility of the adsorption process, inherent to physisorption, suggests potential applications of this system in: (i) controlled/sustained drug delivery platforms, where MET release can be modulated by pH or solvent polarity; and (ii) pharmaceutical effluent treatment, where X7 may serve as a recyclable adsorbent for the removal of MET from wastewater.

These results provide a spectroscopic basis for the further development and optimization of X7-based systems for pharmaceutical applications involving MET.

SEM/EDX analyses showed pore filling and the appearance of N as an elemental marker of MET uptake, while TG/DSC data indicated modified thermal behavior of the loaded resin. XRD patterns confirmed the amorphous nature of X7 and the absence of crystalline MET deposits, supporting the conclusion that MET is dispersed at the molecular level within the polymer matrix.

Regeneration studies demonstrated that aqueous eluents are ineffective (<10%), whereas the (1:1) MeOH–1M HCl system achieved 89.3% desorption efficiency, highlighting the resin’s potential for repeated use in circular treatment schemes. The combination of good adsorption capacity, pH-independent performance, and efficient solvent-assisted regeneration positions X7 resin as a technically viable and sustainable adsorbent for the removal and recovery of highly polar pharmaceuticals.

## Figures and Tables

**Table 1:** Influence of contact time on MET adsorption onto X7.

Morris–Weber (intraparticle diffusion)	k_id_ (mg/g min^−0.5^)	C (mg/g)	R^2^
	5.32	7.43	0.8971
Elovich	α (mg/g∙min)	β (g/mg)	R^2^
	1.91	0.06	0.9700
PFO	k_1_ (g/(mg∙min))	Q_e_ (mg/g)	R^2^
	0.061	76.5	0.9259
PSO	k_2_ (g/(mg∙min))	Q_e_ (mg/g)	R^2^
	0.0003	78.7	0.9881

**Table 2:** Adsorbents used for metformin removal and their adsorption capacities.

Materials Used for Adsorption Studies	Adsorption Capacity (mg/g)	References
Cu/ZnO nanoparticles anchored on carboxylated graphene oxide (GO)	232.56	[53]
Biochars from corn stalk (CSB700), walnut shell (WSB700) and cow manure (CMB700)	16.79, 11.93, and 7.53	[54]
Graphene oxide (GO)	96.7	[30]
Graphene oxide nanoparticles (GO-NPs)	122.61	[31]
Bifunctional Fe-ZSM-5 nano-adsorbent	14.99	[32]
Activated carbon from water hyacinth (H_3_PO_4_-activated)	122.47	[59]
Graphene-based composites: GO, CTOS-mGO, PFPA-rGO	90.9, 95.2, and 91.7	[43]
Silica–alumina composite (SA)	45.7	[44]
Activated carbon from orange peel (acid-activated)	50.99	[75]
Amberlite XAD7HP (X7)	62	This study

**Table 3:** Isotherm-calculated parameters for MET adsorption on X7 resin.

**Langmuir**		**Temkin–Pyzhev**	
Q_o_ (mg/g)	68.5	A (L/mg)	1.26
b (L/mg)	0.063	b_T_ (J/mol)	181.3
R_L_	0.05	B	13.7
R^2^	0.9954	R^2^	0.8839
**Freundlich**		**Dubinin–Radushkevich**	
K_f_ (L/mg)	6.63	q_m_ (mg/g)	46.82
1/n	0.50	β (mol^2^/kJ^2^)	3 × 10^−6^
n	2.01	E (kJ/mol)	4.08
R^2^	0.8549	R^2^	0.8856

**Table 4:** Key FTIR band assignments and modifications.

Band Position (cm^−1^)	Assignment	Amberlite X7	MET	X7-MET	Interpretation
3367/3292	ν N–H asym./sym. (–NH_2_)	Absent	Present, intense	Absent	H-bonding N–H···O=C
3151	ν N–H (HCl salt, aggregates)	Absent	Present, broad	Absent	Disruption of H-bond aggregates
2964 → 2967	ν C–H aliphatic	2964.31	Absent	2967.42	Minor conformational change
1723 → 1723.17	ν C=O ester (Amberlite)	1723.23	Absent	Retained, ↓ rel. intensity	C=O acts as H-bond acceptor
1637 → 1636.72	ν C=C/δ H_2_O	1637.48 (weak)	Absent	Intensified	Overlap with adsorbed MET C=N
1622/1563	ν C=N + δ N–H (biguanide)	Absent	Present, dominant	Absent	Biguanide groups immobilized
1140 → 1139.24	ν C–O–C sym. (ester)	1140.00	Absent	Conserved	Resin structure intact; physisorption
1256	ν C–O–C asym. (ester)	Present	Absent	Conserved	Ester linkage unaffected

## Data Availability

The original contributions presented in this study are included in the article/Supplementary Material. Further inquiries can be directed to the corresponding author.

## Supplementary Materials

The following supporting information can be downloaded at https://www.mdpi.com/article/10.3390/polym18141751/s1: Figure S1: Fitting of adsorption isotherm models of MET on X7: Langmuir (a), Freundlich (b), Dubinin–Radushkevich (c), and Temkin (d). Figure S2: Kinetic model fitting of MET on X7: Morris–Weber intraparticle diffusion model (a), Elovich model (b), PFO model (c), and PSO model (d).
