# Supplementary material for: Towards Circular Water Treatment: Adsorption Mechanism and Analytical Characterization of Metformin Retention on Amberlite XAD7HP Resin
Source: Polymers (Basel). 2026 Jul 17;18(14):1751. doi: 10.3390/polym18141751 (PMC13416504; doi:10.3390/polym18141751)
Supplement: Supplementary file 1 [file polymers-18-01751-s001.zip › polymers-4420315-supplementary.pdf]

polymers

MDPI

Supplementary Material

Towards Circular Water Treatment: Adsorption Mechanism and Analytical Characterization of Metformin Retention on Amberlite XAD7HP Resin

Valentin Romeo Marin [1], Nicoleta Mirela Marin [2,3,4,*], Toma Galaon [2,3], Adriana Mariana Borş [5], Ludmila Motelica [6,7], Otilia Ruxandra Radacina [4], Marian Rascov [4] and Ovidiu Oprea [7,8,9]

1 C.M.V. DR. MARIN ROMEO SRL, 235601 Oraş Scorniceşti, Romania; romeomarinvet@yahoo.com
2 National Research and Development Institute for Industrial Ecology ECOIND, 060652 Bucharest, Romania; tomagalaon@yahoo.com
3 Department of Analytical and Physical Chemistry, University of Bucharest, 030018 Bucharest, Romania
4 Department of Oxide Materials Science and Engineering, National University of Science and Technology POLITEHNICA Bucharest, 060042 Bucharest, Romania; otiliaradacina@yahoo.com (O.R.R.); marian.rascov@yahoo.com (M.R.)
5 National Institute for R&D for Optoelectronics—Subsidiary, Research Institute for Hydraulics and Pneumatics—INOE 2000-IHP, 040558 Bucharest, Romania; bors.ihp@fluidas.ro
6 Research Center for Advanced Materials, Products and Processes, National University of Science and Technology POLITEHNICA Bucharest, 060042 Bucharest, Romania; ludmila.motelica@upb.ro
7 National Centre for Micro- and Nanomaterials, National University of Science and Technology POLITEHNICA Bucharest, 060042 Bucharest, Romania; ovidiu.oprea@upb.ro
8 Academy of Romanian Scientists, 050045 Bucharest, Romania
9 Faculty of Chemical Engineering and Biotechnologies, National University of Science and Technology POLITEHNICA Bucharest, 011061 Bucharest, Romania
* Correspondence: nicoleta.marin@incdecoind.ro

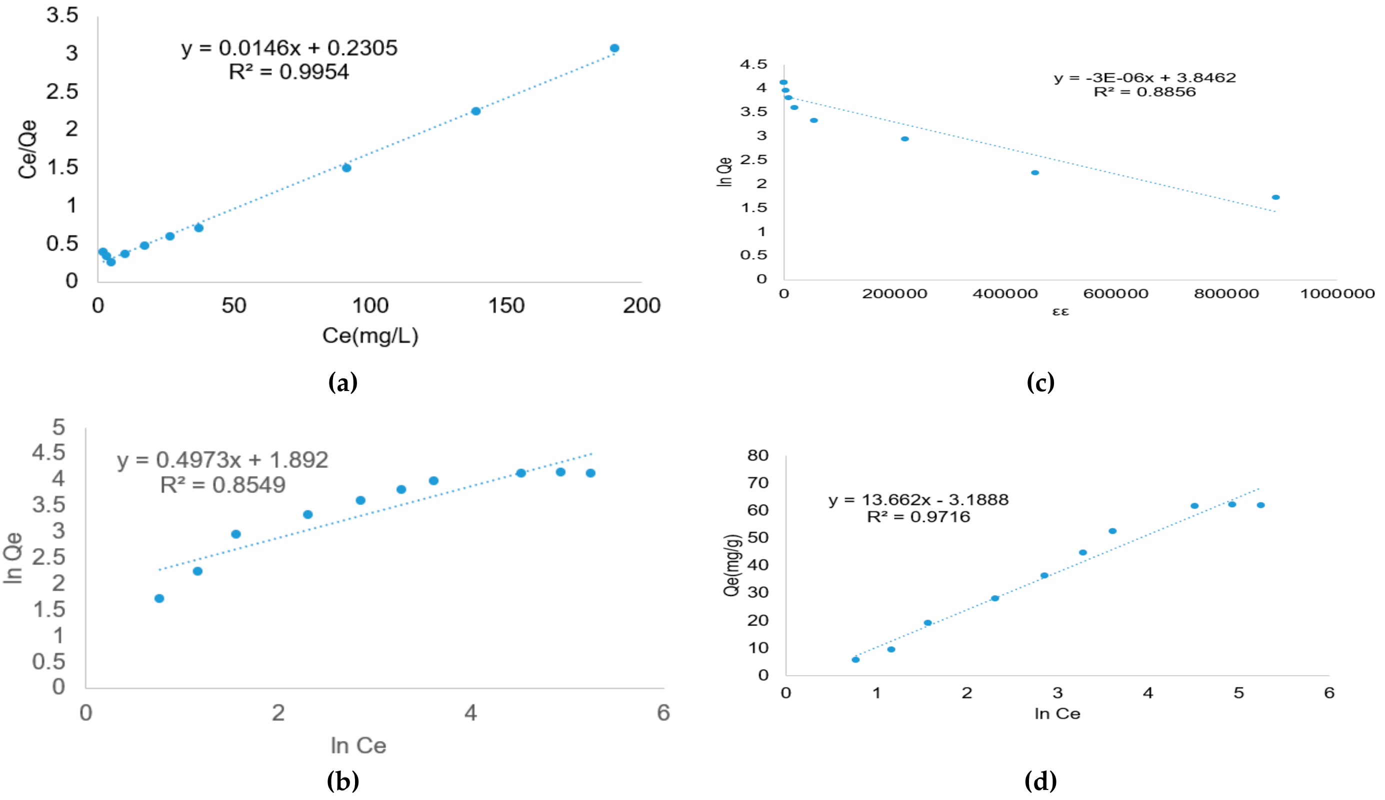

Figure S1. Fitting of adsorption isotherm models of MET on X7: Langmuir (**a**), Freundlich (**b**), Dubinin–Radushkevich (**c**) and Temkin (**d**).

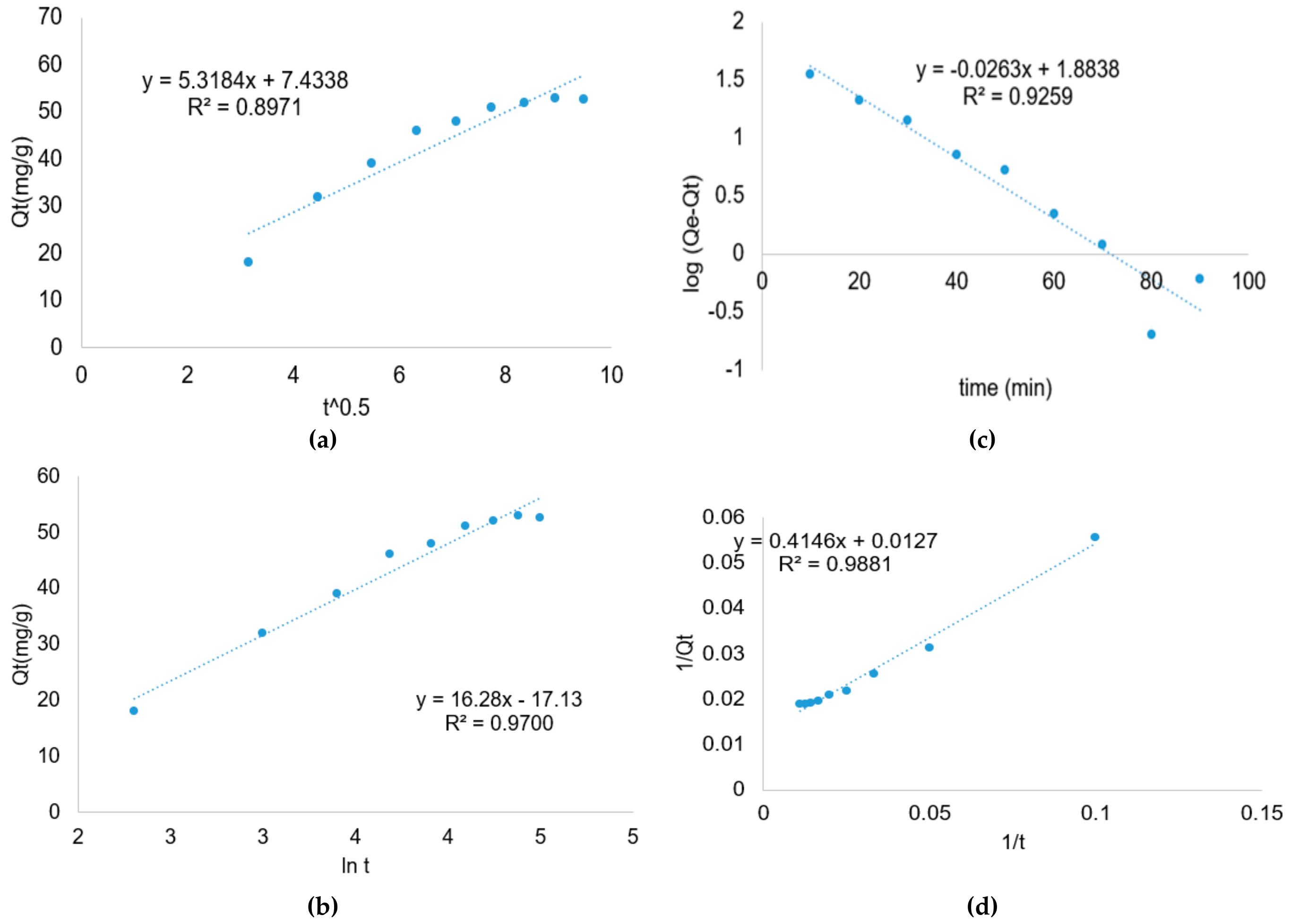

Figure S2. Kinetic model fitting of MET on X7: Weber–Morris intraparticle diffusion model (**a**), Elovich model (**b**), PFO model (**c**), and PSO model (**d**).
